# Escalating *Plasmodium falciparum *antifolate drug resistance mutations in Macha, rural Zambia

**DOI:** 10.1186/1475-2875-7-87

**Published:** 2008-05-21

**Authors:** Mtawa AP Mkulama, Sandra Chishimba, Jay Sikalima, Petrica Rouse, Philip E Thuma, Sungano Mharakurwa

**Affiliations:** 1 The Malaria Institute at Macha, Namwala Road, Choma, Zambia; 2 Johns Hopkins Bloomberg School of Public Health, Baltimore, Maryland, USA

## Abstract

**Background:**

In Zambia the first-line treatment for uncomplicated malaria is artemisinin combination therapy (ACT), with artemether-lumefantrine currently being used. However, the antifolate regimen, sulphadoxine-pyrimethamine (SP), remains the treatment of choice in children weighing less than 5 kg and also in expectant mothers. SP is also the choice drug for intermittent preventive therapy in pregnancy and serves as stand-by treatment during ACT stock outs. The current study assessed the status of *Plasmodium falciparum *point mutations associated with antifolate drug resistance in the area around Macha.

**Methods:**

A representative sample of 2,780 residents from the vicinity of Macha was screened for malaria by microscopy. At the same time, blood was collected onto filter paper and dried for subsequent *P. falciparum *DNA analysis. From 188 (6.8%) individuals that were thick film-positive, a simple random sub-set of 95 *P. falciparum *infections were genotyped for DHFR and DHPS antifolate resistance mutations, using nested PCR and allele-specific restriction enzyme digestion.

**Results:**

*Plasmodium falciparum *field samples exhibited a high prevalence of antifolate resistance mutations, including the DHFR triple (Asn-108 + Arg-59 + Ile-51) mutant (41.3%) and DHPS double (Gly-437 + Glu-540) mutant (16%). The quintuple (DHFR triple + DHPS double) mutant was found in 4 (6.5%) of the samples. Levels of mutated parasites showed a dramatic escalation, relative to previous surveys since 1988. However, neither of the Val-16 and Thr-108 mutations, which jointly confer resistance to cycloguanil, was detectable among the human infections. The Leu-164 mutation, associated with high grade resistance to both pyrimethamine and cycloguanil, as a multiple mutant with Asn-108, Arg-59 and (or) Ile-51, was also absent.

**Conclusion:**

This study points to escalating levels of *P. falciparum *antifolate resistance in the vicinity of Macha. Continued monitoring is recommended to ensure timely policy revisions before widespread resistance exacts a serious public health toll.

## Background

Malaria kills an estimated 0.7 to 2.7 million people, of which 75% are African children [1]. Finding effective drug treatment is a necessity; however, drug resistance is an increasing problem, which poses a great challenge for control. Tanzania first reported emerging SP resistance in Africa around 1994 – 1995 [2,3].

During the period of 2000–2005, Zambia revised its national malaria drug treatment policy to adopt artemisinin combination therapy (ACT) as the standard of care nationally [4] This essentially led to the replacement of chloroquine and sulphadoxine-pyrimethamine (SP) monotherapy with ACT. Nevertheless, SP is still widely used and remains the drug of choice to treat uncomplicated malaria in children weighing less than 5 kg and in pregnant women. SP is also the drug of choice for intermittent presumptive therapy in pregnancy (IPTp) and serves as stand-by treatment during stock-outs of ACT's. Moreover, recent efforts to scale-up malaria control in endemic countries, including increased support for commodities and health systems, is resulting in greater access to and a vastly increased use of antimalarial medicines [4]. This leads to a much higher degree of drug pressure on the parasite which will almost certainly increase the likelihood of selecting for resistant parasite genotypes.

Resistance to antifolate drugs is associated with point mutations in the dihydrofolate reductase domain of the dihydrofolate-thymidylate synthetase (DHFR-TS) gene and dihydropteroate synthase region of the pyrophosphokinase-dihydropteroate synthetase (PPK-DHPS) gene of the malaria parasite. Sulpha drugs act by selectively inhibiting the PPK-DHPS enzyme in the folate pathway of the parasite [5]. The genes encoding the PPK-DHPS and DHFR-TS enzymes have been sequenced in *P. falciparum *and point mutations have been identified which are associated with *in vitro *pyrimethamine-sulphadoxine resistance. Point mutations in the following codons of the DHPS domain are known to confer resistance to sulphadoxine: Ser-436 to Ala-436; Ala-437 to Gly-437; Lys-540 to Glu-540; Ala-613 to Ser-613 or Thr-613. Resistance to pyrimethamine is conferred by point mutations in the DHFR domain, with the associated amino acid changes as Ala-16 to Val-16, Asn-51 to Ile-51; Cys-59 to Arg-59; Ser-108 to Asn-108/Thr-108, and Ile-164 to Leu-164. SP resistance is therefore attributed to parasites that carry point mutations at codons 16, 51, 59, 108 and 164 of the DHFR gene and is further augmented by point mutations at codons 436, 437, 540, 581 and 613 of the DHPS gene [6-8]. The present study assessed the prevalence of DHFR and DHPS mutations in *P. falciparum *isolates from residents of the Macha area in Southern Province of Zambia.

## Methods

### Study area and population

The study was conducted in a 2,000 km^2 ^area around Macha, approximately 75 km north of Choma in Zambia's southern province. The area lies 900 – 1,000 m above sea level and traditionally has experienced hyperendemic malaria transmission. Residents in this community are primarily subsistence farmers of the *BaTonga *tribe.

### Study design and data collection

The study was a prospective cross-sectional survey, based on willing participants of all ages. The area around Macha was divided into 80 geographical grids of 25 km^2 ^apiece, in extent. Using a simple random sampling procedure, ten such grids were chosen, followed by one headman area (an administrative unit comprising an average of 270 residents in a group of households, which report to one elected household head, called a headman) from 2–5 possible per grid. By prior arrangement, following permission from the relevant chiefs and headmen, all willing individuals from each of the ten chosen headmen areas, in turn, assembled at a central location of their choice for malaria screening by microscopy. Thick blood films and filter paper blood spots were collected by finger-prick from a total sample of 2,780 residents. One hundred and eighty-eight (188) samples were thick film-positive. A simple random sub-set of 95 samples were subsequently genotyped for *P. falciparum *DHFR and DHPS antifolate resistance mutations.

### DNA extraction and molecular analysis

Parasite DNA was extracted using the chelex method[9] (chelex 100 resin (Biorad^®^) was employed). Antifolate resistance polymorphisms in DHFR and DHPS genes were detected by nested PCR and allele specific restriction enzyme digestion as previously described [10]. PCR amplicon and restriction fragments were resolved by electrophoresis on 2% ethidium bromide agarose gels and visualized by UV transillumination on a 1D Kodak (EDAS 290) imaging system.

### Ethics

Permission for the study was sought from resident chiefs, headmen, household heads and study participants themselves (or guardians in the case of children). The study was approved by the University of Zambia Research Ethics Committee (UNZA-REC).

## Results

**Figure 1:**
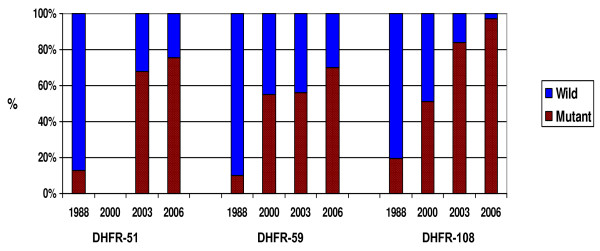
Prevalence of *P. falciparum *antifolate resistance mutations in the vicinity of Macha, by DHFR codon, from 1988 – 2006 (n = 110, 49, 25 and 95, respectively for 1988, 2000, 2003 and 2006).

**Figure 2:**
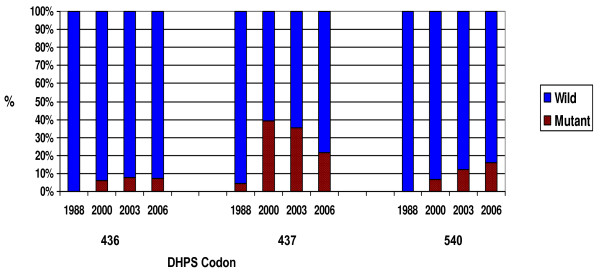
*Plasmodium falciparum *DHPS mutations in the vicinity of Macha, 1988 – 2006 (n = 110, 49, 25 and 95, respectively for 1988, 2000, 2003 and 2006).

Virtually all the *P. falciparum *infections assayed (97.5%) bore the pyrimethamine-resistant DHFR Asn-108 mutation, 17.5% occurring as a mixture with wild type Ser-108. High levels of the DHFR Ile-51 and Arg-59 mutations were also found (Figure 1). Albeit less prevalent, DHPS mutations occurred at codons 436, 437 and 540 (Figure 2).

There were appreciable multiple antifolate resistance mutants, including DHFR triple (Ile-51/Arg-59/Asn-108), DHPS double (Gly-437 + Glu-540) and the quintuple (DHFR triple + DHPS double) mutant (Table 1).

**Table 1:** Antifolate resistance multiple mutants in the vicinity of Macha, 1988 – 2006.

	Mutant			
		
Survey Year	DHFR triple	DHPS double	DHFR/DHPS quintuple	N
1988	0 (0.0%)	0 (0.0%)	0 (0.0%)	110
2000	0 (0.0%)	1 (2.0%)	0 (0.0%)	49
2003	10 (40.0%)	2 (8.0%)	1 (4.0%)	25
2006	38 (41.3%)	10 (16.1%)	4 (6.5%)	95

Antifolate resistance mutations have evidently escalated over the years, since previous surveys (Thuma *et al *unpublished data) in the area from 1988 (Figure 1 – Figure 2, Table 1). The odds of infections carrying Ile-51, Cys-59 and Asn-108, have increased 15× (OR [95% CI]: 14.9 [6.02–36.89], p < 0.001), 11× (OR [95% CI]: 10.6 [5.13–21.98]), and 18× (OR [95% CI]: 18.4 [9.00–37.59]), respectively for 2006 relative to 1988.

Absent among the infections were the DHFR Thr-108, Val-16 and Leu-164 mutations. The DHPS Phe-436, Ser/Thr-613 and Gly-581 mutations were not found, and there were no DHFR quadruple mutants among the field samples.

## Discussion

This study documents the current status of *P. falciparum *antifolate resistance mutations in an area of the Southern Province of Zambia. The results show elevated levels of DHFR mutations, with practically saturated (pyrimethamine-resistant) Asn-108, and multiple mutants setting in.

It is evident that antifolate resistance mutants have been escalating dramatically among local *P. falciparum *infections since 1988. This trend can be expected from drug selection pressure due to SP use over the years. Originally SP was second line medication in Zambia for suspected chloroquine-resistant cases. However, in 2003, SP was adopted as the drug of choice for treatment of children weighing less than 5 kg and pregnant women. The antifolate combination also continues to serve as the stand-by drug treatment during shortages of ACT, and remains the drug of choice for IPTp. Evolution of resistance to SP upon widespread use of the drug has typically developed more rapidly than to chloroquine [11,12]. Hence antifolate resistance will likely continue to intensify under the present conditions.

The rising prevalence of multiple mutants may well point to the beginning of the end for SP *in vivo *efficacy, since these correlate more closely with therapeutic failure. The quintuple mutant is of major concern, although it was only found in 7% of the infections so far. Nevertheless, the right combinations of *P. falciparum *DHFR and DHPS point mutations that can cause clinical resistance to antifolates are already prevalent. These prevailing mutated parasite profiles may explain localized malaria outbreaks that now consistently occur at health centers and clinics during ACT stock-outs, especially when there is reversion to use of SP. Avoidance of ACT shortages at health centers and clinics is, therefore, paramount. Continued molecular surveillance and *in vivo *therapeutic efficacy assessments are recommended to help ensure appropriate policy revisions before widespread therapeutic failure claims a serious public health toll.

## Authors' contributions

MAPM conducted the genotyping of the parasites, data collection and prepared the manuscript. SC did data entry, assisted in lab assays and management. JS assisted in field data collection and conducted microscopy for the study. PR assisted in lab assays and contributed helpful input to the manuscript. PET provided invaluable technical guidance and critical input to the manuscript. SM was the overall project leader who came up with the idea, did the statistical analysis as well as providing technical guidance and critical input on the manuscript. All authors read and approved the final version of the manuscript.
